# Radiomics analysis based on CT’s greater omental caking for predicting pathological grading of pseudomyxoma peritonei

**DOI:** 10.1038/s41598-022-08267-0

**Published:** 2022-03-15

**Authors:** Nan Zhou, Ruixue Dou, Xichao Zhai, Jingyang Fang, Jiajun Wang, Ruiqing Ma, Jingxu Xu, Bin Cui, Lei Liang

**Affiliations:** 1 Department of Ultrasound, Aerospace Center Hospital, Beijing, China; 2 Department of Myxoma, Aerospace Center Hospital, Beijing, China; 3 Department of Research Collaboration, R&D Center, Beijing Deepwise & League of PHD Technology Co., Ltd, Beijing, China; 4 Department of Radiology, Aerospace Center Hospital, Beijing, China

**Keywords:** Cancer, Computational biology and bioinformatics

## Abstract

The objective of this study was to predict the preoperative pathological grading and survival period of *Pseudomyxoma peritonei* (PMP) by establishing models, including a radiomics model with greater omental caking as the imaging observation index, a clinical model including clinical indexes, and a combined model of these two. A total of 88 PMP patients were selected. Clinical data of patients, including age, sex, preoperative serum tumor markers [CEA, CA125, and CA199], survival time, and preoperative computed tomography (CT) images were analyzed. Three models (clinical model, radiomics model and combined model) were used to predict PMP pathological grading. The models’ diagnostic efficiency was compared and analyzed by building the receiver operating characteristic (ROC) curve. Simultaneously, the impact of PMP’s different pathological grades was evaluated. The results showed that the radiomics model based on the CT’s greater omental caking, an area under the ROC curve ([AUC] = 0.878), and the combined model (AUC = 0.899) had diagnostic power for determining PMP pathological grading. The imaging radiomics model based on CT greater omental caking can be used to predict PMP pathological grading, which is important in the treatment selection method and prognosis assessment.

## Introduction

*Pseudomyxoma peritonei* (PMP) is a disease characterized by significant mucus (jelly like material) dispersed in the peritoneum or omentum. In clinical practice, it is a rare condition^1^, and its incidence is approximately 1–2 per million per year^2,3^. Its pathological source and grade have been controversial. According to the *WHO's Classification of Tumors of the Digestive System* (2010), PMP is divided into high and low grades based on the histological arrangement of tumor cells, cell atypia size, and whether there are signet ring cells^4^.

Carr^5^ analyzed 274 PMP cases and found that the survival rate of low-grade PMP was significantly higher than that of high-grade PMP; the overall 5-year survival rates were 63% and 23%, respectively. The follow-up results of Ronnett^6^ showed that the patient-survival rate with low-grade PMP was significantly higher than that with high-grade PMP. The 5-year and 10-year survival rates were 75% and 68%, respectively, and the 5-year and 10-year survival rates for high-grade PMP were 14% and 13%, respectively. Ross^7^ found that patients with disseminated peritoneal adenomucinosis had a better three-year survival rate than patients with high-grade appendiceal tumors. The researchers concluded that the survival rate of low-grade PMP was significantly higher than that of high-grade PMP.

The above studies highlight that pathological grading is an independent factor that affects patient survival and mortality with PMP^8^. The preoperative pathological grading has a direct impact on the patient's treatment and prognosis. Therefore, it is of great significance to clinician if the pathological grading can be assessed through preoperative examination.

Presently, computed tomography (CT) is an effective imaging method for the preoperative diagnosis of PMP. Studies have shown that greater omental caking is one of PMP’s primary imaging manifestations and has important value in its diagnosis^9,10^, but there is no relevant report on its predictive performance in PMP pathological grading. In the early stage of tumorigenesis development, some special molecular markers in serum, such as CEA, CA199, CA125, and CA724, may increase to varying degrees. Due to the advantages of convenient operation and basic noninvasiveness in the detection process, these serum tumor markers are currently common indexes for clinical disease screening, recurrence monitoring, and prognosis evaluation^11–15^. Among them, CEA, CA199, and CA125 are commonly used indicators for PMP preoperative screening, but they lack certain specificity for the disease’s diagnosis and are mostly used in recurrence monitoring.

The study purpose was to establish models, including radiomics models with CT greater omental caking, clinical models that contained some associated molecular marker clinical indicators, and a combination of the two models in an attempt to prompt the preoperative pathological grading and prediction of PMP survival period.

## Research methods

### Research object

**Figure 1:**
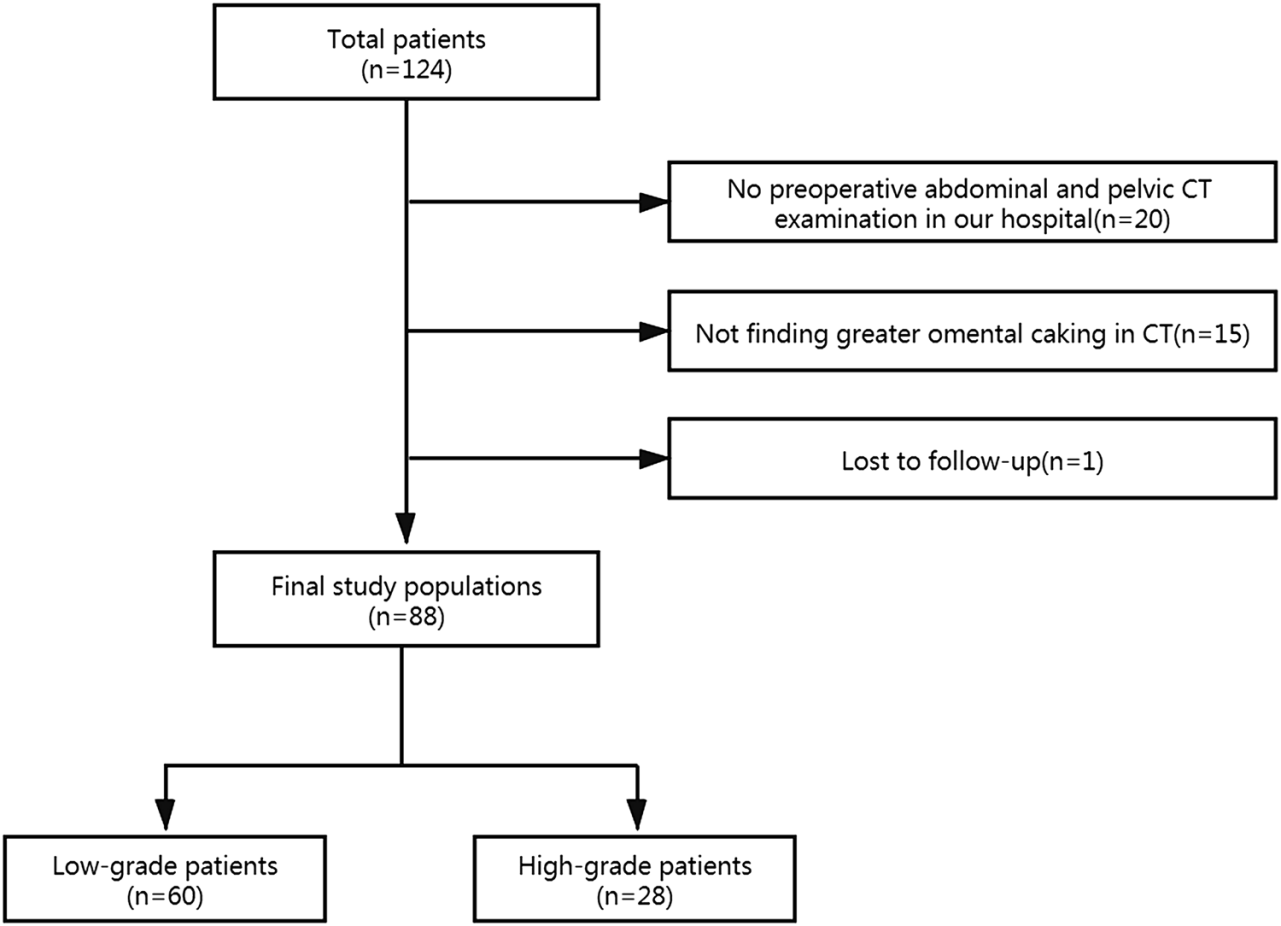
Patient selection flow chart.

This research was approved by the ethic Committee of Aerospace Central Hospital, and informed consent was signed by all patients, and all methods were performed in accordance with the relevant guidelines and regulations. In total, 124 patients with PMP from January 2014 to January 2019 from Aerospace Central Hospital were included continuously. Patients were included by criteria as follows: (1) patients were diagnosed with PMP by surgery and pathology in our hospital, (2) no tumor reduction surgery was performed, (3) patients weren’t given neoadjuvant therapy. Patients were excluded by criteria as follows: (1) patients who did not undergo preoperative CT examination of the abdominal and pelvic cavity in our hospital (n = 20), (2) patients without greater omental caking (n = 15), and (3) patients lost to follow-up (n = 1). Ultimately, a total of 88 patients were selected, including those with 60 low-grade PMP and 28 high-grade PMP. Figure 1 shows the details of patient selection.

### Pathological results

All patients underwent cytoreductive surgery and hyperthermic intraperitoneal chemotherapy (CRS-HIPEC) surgery in our hospital, and postoperative pathological results were obtained.

### Clinical data

All patients’ relevant clinical data were collected, including age, sex, preoperative serum tumor markers, including CEA, CA-125, CA-199, and survival time. In the follow-up, tumor markers were evaluated, and CT was performed every six months after surgery. Those who were unable to go to the hospital for check-up were followed-up by telephone. The follow-up time for this study ended on February 1, 2020. If death occurred during the follow-up, the follow-up time ended at the time of death.

### Image and data

**Figure 2:**
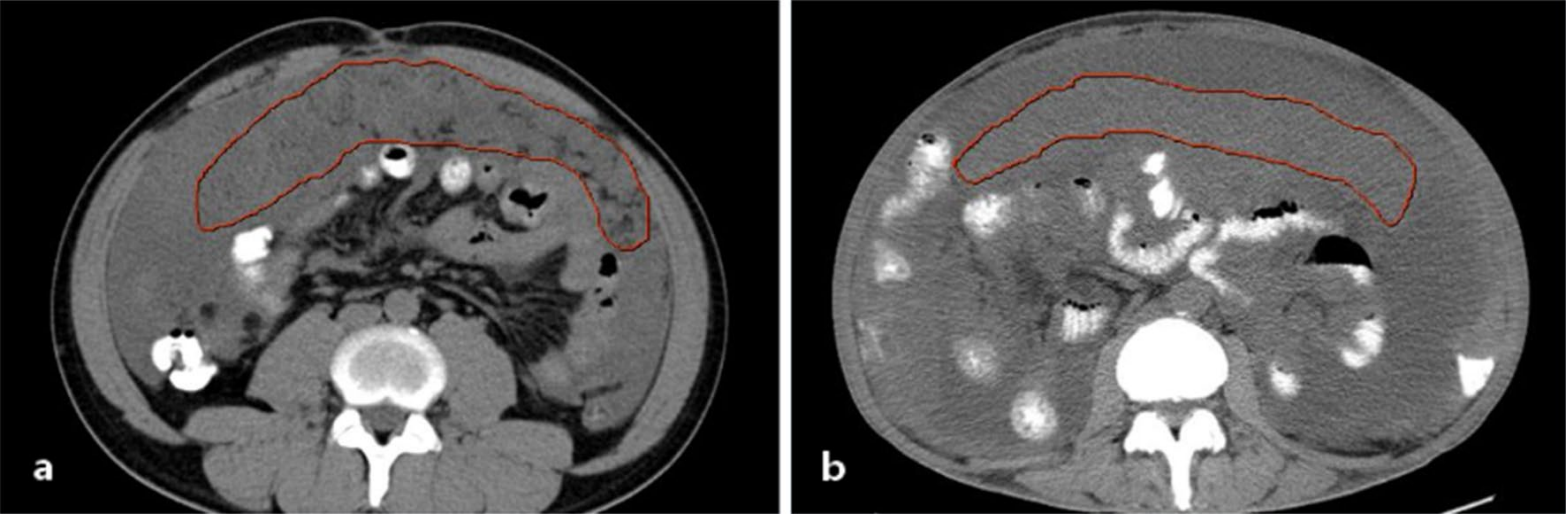
The CT ROI area of the greater-omental caking. (**a**) A 37-year-old man with low-grade PMP; the red curve shows greater omental caking. (**b**) A 62-year-old woman with high-grade PMP; the red curve shows greater omental caking.

All patients had a plain abdominal and pelvic CT scan before the operation using 64-slice spiral CT (Siemens Healthcare, Germany) with a thickness of 5 mm, an interval of 5 mm, and a pitch of 1 to 1.2. All patients were prepared for gastrointestinal tract before examination: fasting and water deprivation for 8 h. CT images were stored in DICOM format. The images were imported into ITK-SNAP software (version 3.8.0), and the tumor boundary as manually produced to determine the tumor region of interest (ROI). The thickening of the omentum which is recognized by CT was defined as omental caking. The standard of tracing greater omental caking was used to select the image that could display the largest greater omental caking; the ROI area enveloped the greater omental caking, and the unit was square centimeter. The ROI area of greater omental caking is shown in Fig. 2. All cases were retrospectively studied by two radiologists, working independently. Meanwhile, the accuracy of lesions and relevant data were inspected by a specialist.

### Radiomics feature extraction

The Dr. Wise Multimodal Research Platform (https:/keyan.deepwise.com; Beijing Deepwise & League of PHD Technology Co., Ltd, 193 Beijing, China) was used for the CT’s feature extraction. Approximately 1218 features were extracted from the CT lesion’s ROI extracted and categorized into seven categories: first order features, shape based features, gray-scale co-occurrence Matrix (GLCM) features, gray-level size zone matrix (GLSZM) features, gray-level run length matrix (GLRLM) features, gray-level distance-zone matrix (GLDM), and neighboring gray level dependence matrix^16^.

The results of training sets and validation sets were tested by the fivefold cross validation method. To study the correlation between clinical factors and greater omental caking, logistic regression models were deployed. To build the radiomics model, and six kinds of feature-screening methods were used: those that included the F-Test, Pearson Correlation Coefficient, Mutual Information, L1-based models, tree-based models, and Recursive Feature Elimination. We only used one of these methods each time in model construction. Finally, a method based linear models penalized with the L1 norm was selected for feature screening and it had the best diagnostic performance. RAD-SCORE was generated by analyzing logistic regression weighted by their coefficients of the features. We used multivariate logistic regression to construct the clinical-radiomics combined model; meanwhile, it was presented in the nomogram chart^16^.

### Statistical analysis

Univariate logistic regression and multivariate logistic regression were used to establish the clinical model. Then, the backward stepwise selection method was applied. In evaluation of each model’s performance, we utilized the area under the curve (AUC) with 95% confidence intervals (95% CIs). The DeLong test was used to determine whether all the models’ AUC values were significant. Finally, the nomogram figure of the clinical-radiomics model was established. To confirm the clinical usefulness of the three models, decision curve analysis was conducted. A Cox regression model with the Kaplan–Meier method was used for survival analysis. R software (version 4.0.2) and SPSS (version 23.0) were applied for analysis.

### Ethics statements

Studies involving animal subjects.

### Generated statement

No animal studies are presented in this manuscript.

### Studies involving human subjects

Generated Statement: The studies involving human participants were reviewed and approved by the Ethics Committee of Aerospace Center Hospital. The patients provided written informed consent to participate in this study.

### Inclusion of identifiable human data

Generated Statement: No potentially identifiable human images or data are presented in this study.

## Results

### Patient characteristics

Of the 88 patients, 60 (68%) presented low-grade PMP, and 28 (32%) were diagnosed with high-grade PMP. The primary sites of 88 patients enrolled in our study are the appendix. Table 1 shows all patient features.

**Table 1:** Characteristics of all patients and logistic analysis results of clinical factors.

Characteristics	Low-grade	High-grade	Univariate logistic analysis		Multivariate logistic analysis	
			p value	OR (95% CI)	p value	OR (95% CI)
No.of patients, n	60	28				
**Gender, n**			0.356	0.650 (0.261–1.622)	0.256	0.535 (0.182–21.573)
Male	32	12				
Female	28	16				
Age, y	58.3 ± 11.3	57.4 ± 13.6	0.818	0.995 (0.957–1.036)	0.982	1.001 (0.955–1.048)
CEA (ng/ml)	154.9 ± 224.8	121.9 ± 205.2	0.543	0.999 (0.997–1.002)	0.300	0.999 (0.996–1.001)
CA-199 (U/ml)	554.5 ± 1682.8	1527.9 ± 3551.6	0.104	1.000 (1.000–1.000)	0.225	1.000 (1.000–1.000)
CA-125 (U/ml)	111.5 ± 111.0	211.7 ± 230.8	0.006*	1.006 (1.002–1.011)	0.012	1.006 (1.001–1.011)
Median survival (range), d	750(90–2040)	615(30–2100)				

### Clinical model

Among the clinical indicators, including sex, age, and preoperative serum tumor markers (CEA, CA199, and CA125), CA125 was an important factor that prompted the pathological grading of PMP (P = 0.026), as shown by logistic regression analysis in Table 1. To establish a clinical model with CA125, the AUC of training set was 0.64, and the accuracy rate, sensitivity, and specificity were 0.63, 0.43 and 0.73, respectively. The AUC of validation set was 0.62, and the accuracy rate, sensitivity, and specificity were 0.63, 0.43 and 0.73, respectively.

### Radiomics model

In the training set, the 1218 features were reduced to 20 related ones after applying the LASSO algorithm, then the RAD-SCORE was obtained. The weighted features’ sum is shown in the Appendix. The training set’s AUC value was 0.89, and the accuracy rate, sensitivity, and specificity were 0.83, 0.75, and 0.87, respectively. In the validation set, the AUC value was 0.88, and the accuracy rate, sensitivity, and specificity were 0.82, 0.75 and 0.85, respectively.

### Clinical-radiomics combined model

**Figure 3:**
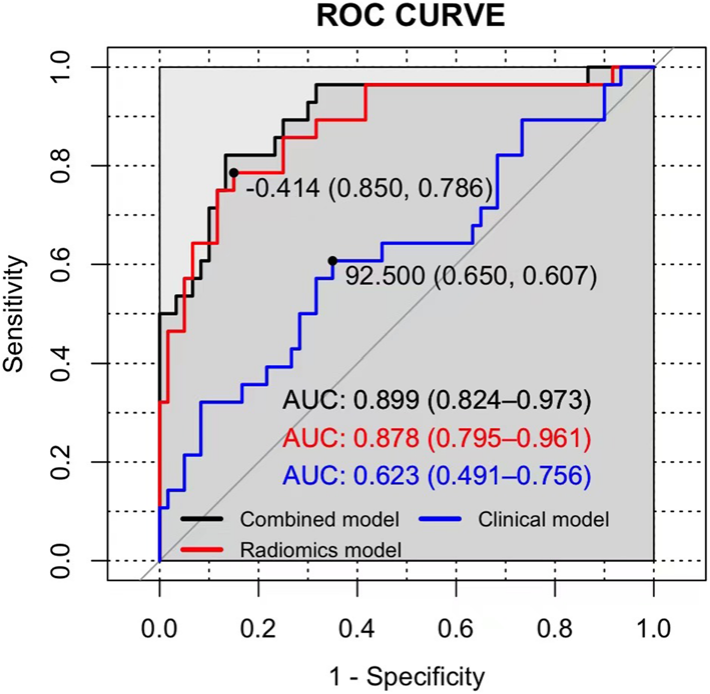
Comparison of ROC curves for the three models’ differentiation for predicting pathological grading.

**Figure 4:**
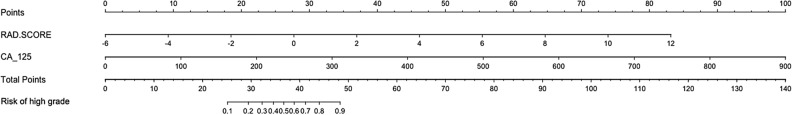
Nomogram of the combined model for predicting the risk of high pathological grade.

The combined clinical-radiomics model showed the best diagnostic performance, with an AUC of 0.92 in the training test and 0.90 in the validation test, respectively. The AUC, accuracy, sensitivity, and specificity of the three models are listed in Table 2. The ROC curves are presented in Fig. 3. The nomogram of the clinical-radiomics combined model is displayed in Fig. 4. From the ROC curves, the cut-off values were got in Fig. 3. The cut-off value of radiomics model is − 0.414 and the corresponding nomogram value is 25 points. The cut-off value of clinical model is 92.5 and the corresponding nomogram value is 10 points. So that, the cut-off value of nomogram is 35 points.

**Table 2:** AUC results of the combined clinical, radiomics, and clinical-radiomics models for predicting the pathological grading of PMP.

	Clinical model		Radiomics model		Clinical-radiomics combined mode	
	Training set	Validation set	Training set	Validation set	Training set	Validation set
AUC	0.64	0.62	0.89	0.88	0.92	0.90
(95% CI)	(0.505–0.764)	(0.491–0.756)	(0.812–0.972)	(0.505–0.764)	(0.505–0.764)	(0.505–0.764)
Accuracy	0.63	0.63	0.83	0.82	0.83	0.83
Sensitivity	0.43	0.43	0.75	0.75	0.75	0.75
Specificity	0.73	0.73	0.87	0.85	0.87	0.87

### Decision curve

**Figure 5:**
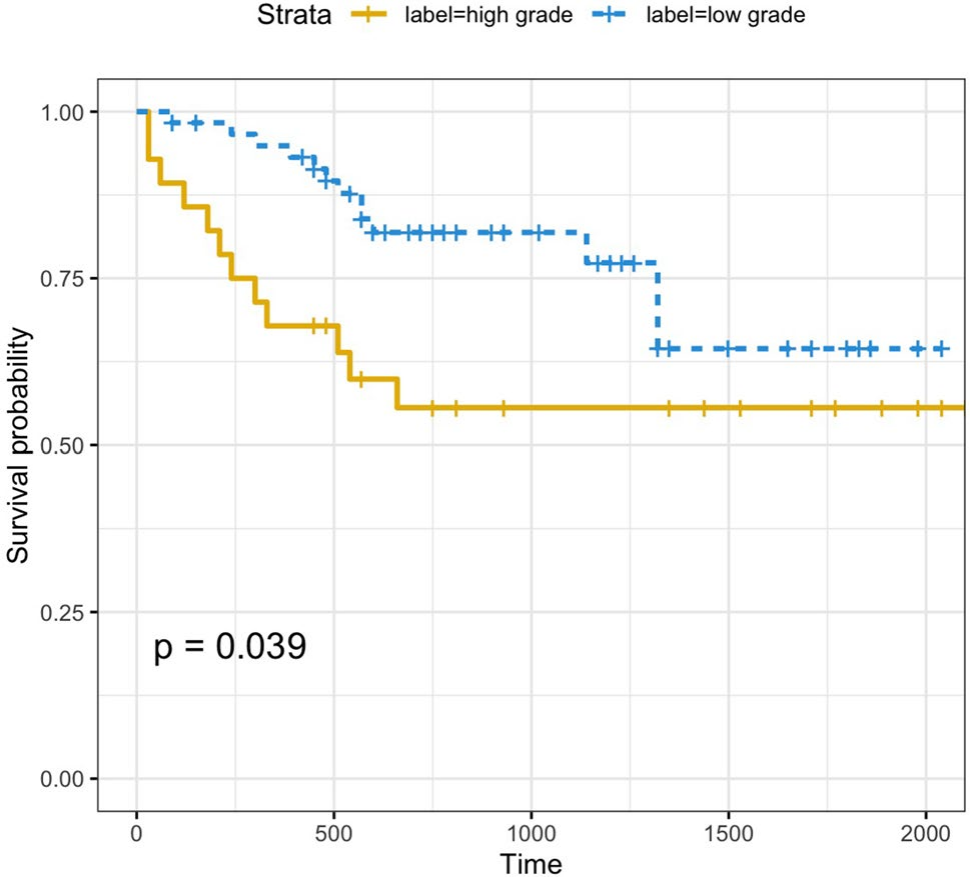
Decision curves of the combined clinical, radiomics, and clinical-radiomics models for predicting pathological grading.

The decision curves of the three models are presented in Fig. 5. From the decision curve, the clinical-radiomics combined model was more beneficial than other models in predicting pathological grading.

### Survival time

**Figure 6:**
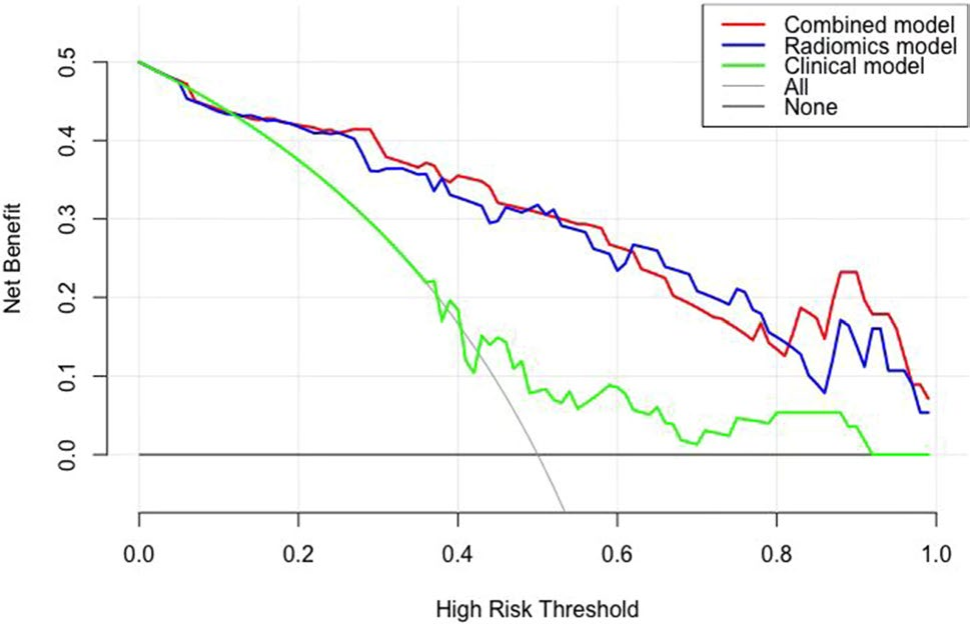
The survival probability of high-grade PMP and low-grade PMP.

In this study, the follow-up time was 0–2000 days; the median follow-up time for high-grade PMP was 615 days, and the median follow-up time for low-grade PMP was 750 days. There was a significant difference in the follow-up time between the high-grade and low-grade PMP groups (P = 0.039). The survival rate curve is shown in Fig. 6.

## Discussion

PMP is a rare clinical entity that is characterized by a significant amount of mucus-jelly substance that widely spreads and settles in the abdominal cavity. The onset of the disease is insidious; the clinical symptoms are atypical, and early diagnosis is difficult. For the differential diagnosis of PMP pathological grading, the sensitivity of physical examination and imaging examination is not high.

Previous studies have indicated that CT identification accuracy and quantification of various peritoneal surface malignancies vary in different primary tumors of origin^17,18^. Presently, CT is also commonly used as a routine preoperative imaging examination method for PMP. Multiple studies have shown that typical imaging manifestations of PMP include mucinous ascites, liver or spleen scallop pressure, peritoneal thickening, greater omental caking and abdominal masses^19,20^. To preoperatively assess the extent of PMP lesions, the utilization of CT has been reported^21^. However, previous CT imaging studies were limited to morphological studies of the disease, which is valuable for diagnosis. However, no relevant reports had suggested pathological grade through CT imaging. Hotta M, et al^22^ found patients with high-grade PMP showed significantly higher PET-PCI and SUVmax than patients with low-grade PMP.

Our research center collected a total of 124 PMP patients from 2014 to 2019. By analyzing the characteristics of the patients’ CT images, we found that 88% of PMP patients had greater omental caking. Studies have shown that the omentum is composed of two layers of peritoneum, which contains blood vessels, lymphatic vessels, lymph nodes, and fatty tissue. The omentum is not only a barrier to disease spread but also a pathway. Hence, the omentum is vulnerable to disease invasion^23^. Tumor cells with PMP exocrine function are widely planted in the peritoneum, causing a significant amount of mucus to accumulate in the abdominal cavity. The mucus protein content is high, and the mucin/cell ratio can be as high as 1:1000, which is relatively viscous^24^. Long-term stimulation of the greater omentum can easily cause fibrosis and the formation of greater omental caking. Greater omental caking is a common imaging feature of PMP, which is one of the reasons why we chose it as a radiomics analysis. Additionally, previous studies have shown that CT is more sensitive and accurate for detecting 0 zone lesions of PMP, which are entire transverse colon and greater omentum lesions^25^. Therefore, we selected greater omental caking as a target of radiomics analysis.

Some studies have shown that the pathological manifestation of jelly like mucus in the abdominal cavity of PMP is simple acellular mucus. Its density, which is the CT value, is higher than that of conventional ascites and other cystic lesions but lower than the soft tissue density. The cell-free mucus material showed a low-density area in a plain CT scan^20^. Different grades of PMP have similar clinical symptoms and similar imaging manifestations on plain CT scans. Traditional imaging can only provide general information, such as tumor size, shape, or density, and has limited value for distinguishing the pathological grading of PMP. Radiomics transforms the quantitative features in high-through put extracted images into data information and establishes predictive models, which can microscopically reflect tumor heterogeneity and are closely related to tumor histological components and the degree of differentiation. Hence, radiomics provides is effective in the differential diagnosis^26^. Radiomics provided a new opportunity to analyze whether the mucus content in PMP greater omental caking was related to its pathological grade and whether the mucus content could be reflected by CT radiomics analysis to predict the pathological grading.

The study results showed that the characteristics of the greater omental caking CT images were significantly different between high-grade and low-grade PMP. Among them, wavelet-HHL_gldm_Small Dependence Low Gray Level Emphasis (SDLGLE), which measures the small dependence’s joint distribution with lower gray-level values, was higher in low grade, indicating that the low-density areas in the low-grade PMP’s greater omental caking were more dispersed and corresponded to histopathology; it stated that mucus components were more widely distributed, and cells and fiber components were less in intensity. Additionally, the parameter wavelet-HHH_glszm_Gray Level Non-Uniformity (GLN), which measures the variability of the gray-level intensity values in the image, had a lower value in the low-grade PMP, indicating poor uniformity of the low-grade PMP’s greater omental caking in histopathology, and there were more mucus components inside the lesion and fewer cells and fibers. Wavelet-HLL_glszm_SmallAreaLowGrayLevelEmphasis (SALGLE), which measures the proportion in the image of the joint distribution of smaller size zones with lower gray-level values, was higher in low-grade PMP, suggesting that there were more low-density areas in the low-grade PMP’s greater omental caking, and its pathology showed that there were more mucus components and fewer cells and fibers.

The radiomics model of this study showed that there were more low-density areas of the low-grade PMP greater omental caking; there were more mucus components, and there were fewer cell and fiber components. The greater omental caking radiomics features showed significant differences between high-grade and low-grade PMP. The analysis showed that this was related to the pathological and cytological differences between those two. In low-grade PMP, the secretory function of tumor epithelial cells was strong, and a large number of mucus vacuoles could be seen in columnar cells, while in high-grade PMP, the cells were severely dysplastic, which severely damaged the columnar secretory function of the cell, thus reduced cellular mucus^27^.

In addition, this study found that serum CA125 levels were correlated with different pathological grade. In this study, the patient’s sex, age, preoperative serum tumor markers (CEA, CA199, and CA125), and other relevant clinical data were collected to establish a clinical model. It was found that serum CA125 was meaningful in PMP pathological grading. The clinical model had a sensitivity of 0.43 and a specificity of 0.73, indicating that the serum CA125 level had value in predicting PMP’s different pathological grading.

In 1981, Bast obtained the monoclonal antibody OC25 from papillary serous cystic ovarian cancer. The tumor-associated antigen recognized by this antibody was CA125^28^. In 2001, the gene CA125 was cloned and named MUC16^29^; CA125 is a large transmembrane glycoprotein that exists in normal peritoneal mesothelial cells and Mullerian epithelial cells^30,31^. According to the literature reports, serum CA125 levels in humans are simultaneously affected by CA125 produced by primary tumor cells and peritoneal mesothelial cells. Because of cell connections and the basement membrane, it cannot enter the blood circulation, so normal people’s serum CA125 levels are low. PMP’s typical clinical manifestations are mucinous ascites and extensive peritoneal implantation^2,32^. Under influence of malignant tumors, the normal cell membrane barrier is destroyed, leading to the release of CA125 into the blood. The upregulation of the MUC16’s expressionalso causes increase of the serum CA125 level^33^. Additionally, recent studies have shown that CA125 participates in the process of peritoneal implantation by mediating the adhesion of free tumor cells in the abdominal cavity and mesothelin on the surface of peritoneal mesothelial cells^34,35^. High-grade PMP is rich in peritoneal mesothelial cells, so its serum CA125 level is higher, while low-grade PMP is the opposite. In Ross's study, CA125 was the only marker to predict complete cytoreduction independently and to be associated with long-term survival. Patients with elevated CA125 had a lower 3-year survival rate^7^. However, CA125 is also affected by peritonitis, ovarian swelling and so on^29^. Therefore, preoperative serum CA125 levels and different pathological grade have some correlation.

CEA is a broad-spectrum tumor marker, and the detection of its level through continuous follow-up can reflect the curative effect and prognosis of malignant tumors after surgery. CA199 is a glycoprotein tumor antigen. It is a gastrointestinal tumor-related antigen. In our study, we also tried to identify the pathological grading of PMP by CEA and CA199. These tumor markers were also beneficial in the prognosis assessment, and a normal level indicated an improved prognosis^36^. SHIGEKI^37^ analyzed 156 PMP cases and concluded that optimal cutoffs were 18 ng/ml for CEA and 89 U/ml for CA199. The AUCs-ROC were 0.68 and 0.69 for CEA and CA199. However, the Ross study concluded that there was no statistically significant association between elevated CEA, CA199, and tumor histology^7^. Theses results were consistent with the results of our study. The significance of CEA and CA199 are controversial in predicting pathological grading of PMP before surgery.

In our study, the combined clinical-radiomics model showed the best diagnostic efficacy by comparing the ROC curves. According to the decision curve, the clinical-radiomics combined model was more beneficial in a wide range of high risk threshold than the clinical and radiomics models alone in predicting high grade PMP. The cut-off value of nomogram is 35 points. That is, nomogram higher than 35 points indicates high-grade PMP, and nomogram lower than 35 points indicates low-grade PMP. Improvement of accuracy in models combining radiomics with clinical factors has previously been reported in many studies^16,38^.

The survival curve shows that the survival rate of low-grade PMP is higher than that of high-grade PMP. This result is related to PMP’s pathological basis. The internationally recognized PMP pathological grading is mainly based on the number of tumor cells and the shape of cancer nests, cell atypia, mitotic figures, and surrounding forms of invasion^32^. The pathological feature of low-grade PMP is mucus. There are many pools and few tumor epithelial cells in the mucus. The tumor cells are columnar or flat and arranged in a single layer or in the form of cords, islands, and clusters. Tumor cells are mildly atypical, and the nuclei are small and regular. There is push-like infiltration of other organs. The pathological features of high-grade PMP are abundant tumor epithelial cells, which can be arranged in strips, islands or cribriform; tumor cells are severely atypical (at least focally atypical), with large nuclei, obvious nucleoli, and vesicular chromatin. Mitotic figures are more common and can destructively infiltrate the internal organs of the abdominal cavity and significantly promote the fibro interstitial response^39^. Low-grade tumors have optimal cytological characteristics, while high-grade tumors have many cells and high heterogeneity. Choudry^40^ retrospectively analyzed the clinicopathological data of 310 PMP patients who underwent CRS-HIPEC and divided the patients into 4 groups according to the tumor epithelial cell density: acellular mucus, low-cell density (< 2%), medium-cell density (2% to 19%), and high-cell density (> 20%). The results showed that compared to patients with acellular mucus, PMP patients with medium-cell or high-cell density had a shorter progression-free survival period. Horvath^41^ also obtained similar results. The above two studies suggested that with the increase in tumor cell density, PMP patients had stronger tumor biological activity and a higher risk of disease progression. The biological behavior of low-grade tumor pushing/expanding infiltration was more benign than invasive/destructive infiltration^32^. The biological behavior of high-grade tumors is often more malignant; the tumor tissue quickly proliferates, and it easily extensively involves the surrounding organs, making it difficult to easily remove and remetastasize^42^. Therefore, the survival probability of high-grade PMP is lower than that of low-grade PMP.

This study has the following advances: (1) Previous CT imaging studies were limited to PMP morphological studies. This study is the first report on PMP pathological grading through CT imaging. (2) This study used CT radiomics characteristics and a radiomics model, which were different from the indexes of most studies. Our study proved that combining RAD-SCORE and a clinical model can improve the diagnostic accuracy and provide clinical benefit of CT for PMP pathological grading. (3) The nomogram of the clinical-radiomics combined model shows several disease risk elements, and it presents a risk score for predicting prognosis, which is more convenient and distinct.

There were a few limitations for this study. (1) This study only included the characteristics of greater omental caking on plain CT scanning with the thickness of 5 mm. In the future, it will be useful to add thin section scanning and enhanced CT features for multimodal studies. (2) This study was performed with a small sample size at a single institution. In the future, it will be valuable to obtain more evidence by multicenter verification with a larger sample size. Due to the sample size, a small survival difference between patients with abnormal and normal CEA and CA199 levels was not detected. As the data continue to accumulate, further analysis needs to be implemented. (3) Previous studies have proposed several CT imaging characteristics of PMP, including mucinous ascites, greater omental caking, abdominal mass, etc. This study only selected greater omental caking, and comprehensive evaluation of other features can be added in the future.

## Summary

In this study, a radiomics model based on CT greater omental caking was established to predict PMP pathological grading, and the results proved that this model has high diagnostic efficiency. Additionally, the nomogram can be used to predict PMP pathological grading by integrating image characteristics and clinical indicators. The chart is simple, intuitive, and easy to grasp, which can enhance imaging physicians’ diagnostic confidence; clinicians can receive valuable guidance with a certain degree of preoperative prediction of PMP at the pathological level and specificity of treatment methods.

## Supplementary Information

Supplementary Information.

## Data Availability

The raw data supporting the conclusions of this article will be made available by the authors, without undue reservation.
