# Supplementary material for: Radiomics analysis based on CT’s greater omental caking for predicting pathological grading of pseudomyxoma peritonei
Source: Sci Rep. 2022 Mar 15;12:4401. doi: 10.1038/s41598-022-08267-0 (PMC8924207; doi:10.1038/s41598-022-08267-0)
Supplement: Supplementary file 1 — Supplementary Information. [file 41598_2022_8267_MOESM1_ESM.doc]

Appendix

-0.4977+LLL_firstorder_RootMeanSquaredx0.7777+wavelet-HHL_gldm_SmallDependenceLowGrayLevelEmphasisx0.698+wavelet-LLH_glszm_SizeZoneNonUniformityNormalizedx0.665+wavelet-HHH_glszm_GrayLevelNonUniformityx0.6134+logarithm_glrlm_ShortRunLowGrayLevelEmphasisx0.5727+wavelet-LHH_glrlm_GrayLevelNonUniformityNormalizedx0.5683+wavelet-HHL_glszm_GrayLevelNonUniformityx0.5342+squareroot_ngtdm_Strengthx0.4572+wavelet-LHL_glrlm_LongRunLowGrayLevelEmphasisx0.4395+wavelet-LHH_glszm_SizeZoneNonUniformityNormalizedx0.3544-wavelet-HLL_glszm_SmallAreaEmphasisx0.1696-gradient_firstorder_Minimumx0.2058-wavelet-HLL_firstorder_Kurtosisx0.2244-wavelet-HHL_firstorder_Medianx0.3387-wavelet-HHL_firstorder_RootMeanSquaredx0.3839-squareroot_glcm_InverseVariancex0.3941-exponential_firstorder_Skewnessx0.4742-wavelet-HLL_glszm_SmallAreaLowGrayLevelEmphasisx0.5143
